# Cultural background and diversity: N’golo and Capoeira in play

**DOI:** 10.3389/fpsyg.2025.1545060

**Published:** 2025-05-13

**Authors:** Lívia de Paula Machado Pasqua, Eliana de Toledo

**Affiliations:** ^1^Laboratory of Capoeira – LABCAPO, School of Physical Education and Sports, Fight Department, Federal University of Rio de Janeiro, Rio de Janeiro, Brazil; ^2^Laboratory of Research and Experiences in Gymnastics – LAPEGI, Sports Sciences, School of Applied Sciences, University of Campinas, Limeira, Brazil

**Keywords:** Capoeira, N’golo, cultural diversity, ancestry, African diaspora, inclusion, interpersonal relationships, history of sport

## Abstract

**Introduction:**

Capoeira is an Afro-Brazilian polysemic cultural manifestation, commonly known as fight-game formerly practiced only in streets and restricted environments and currently in training centers, gyms, and mainly in schools. The main question is: how this practice that was marginalized, prohibited by the Brazilian Penal Code in the past, can today promote an improvement in interpersonal relationships and cultural diversity among people?

**Methodology:**

This is a documentary research characterized by a historical approach, based on capoeira*-body* to understand the body narrative. Sources: analysis of N’golo drawings by the artist Neves e Sousa in Galeria Verney (Portugal).

**Results and discussion:**

Categories established *a priori*: *1. characterization of body practice, 2. musicality* and *3. body elements.* In short, we argue that understanding of the African roots in Capoeira is an instrumental for promoting inclusion and representation, highlighting Cultural Diversity in Physical Education as well in people daily life, in different practical contexts of teaching, all over the world.

## 1 Opening the *roda* (introduction)

Opening the *roda*^1^ (Capoeira circle) means starting the game. This study will primarily explore an approach between Capoeira fundamentals (historical, bodily knowledge) and Cultural Diversiust promotion in Sports in order to understand its ability to unite individuals from various cultural backgrounds and how it has the potential to reduce cultural disparities. As can be seen in global newspapers and magazines throughout the time, this manifestation is also present in areas of social vulnerability and refugee camps, with the aim of reducing differences and promoting harmony between people, as exemplified in the reports “Capoeira brings fun and peace to refugees in the Democratic Republic of Congo” (United Nation High Commissioner for Refugees – Unhcr Brazil, 2014) and “Capoeira unites Israelis from across the country” (I24News English, 2023).

Capoeira has a very unusual trajectory, from crime to heritage (Pasqua et al., 2023), a practice that emerged from enslaved workers since colonial times, when people removed from Africa were brought to Brazil, where they recovered and reinvented their knowledge. For this reason, it was also formerly called the *slave game* or *war dance* (Tavares, 2012). Capoeira was severely repressed in imperial times and prohibited by the Brazilian Penal Code (1890); people only practiced in a hidden way. De Araújo (1997) defends a theory of ‘multiple transformations of Capoeira,’ which greatly assists the framing of this modality in different scopes, contributing to the emergence of different expressive forms of Capoeira, mainly those of a ludic nature and sporting, the latter influenced by the ideas of modern sport widespread during the course of the 19th century. It was recognized as a Brazilian Intangible Cultural Heritage by Instituto do Patrimônio Histórico e Artístico Nacional (2008) and World Intangible Cultural Heritage by United Nations Educational, Scientific and Cultural Organization (2014).

In pursuance of illustration and comparison with other sports, Pasqua et al. (2020) conducted a study on the relationships between Capoeira and Gymnastics (Gymnastics for All), in which they identified and analyzed possible relationships of consonance and dissonance between them. In the first moment, the difference in the migratory processes that involved the conception of Capoeira (forced migration and slavery process; recovery and reinvention of ancestral African practices) and the development of Gymnastics (European immigration and resignification of different gymnastic practices) in Brazil was highlighted. For today, the study (Pasqua et al., 2020, p. 165) concluded that, despite different historical trajectories:

*[..] have in common the possibility of expression in the artistic field, pointing to a broader exploration of the manifestation of Capoeira for the creation of artistic compositions, not only with an addition of specific movement of this practice, nor with a berimbau sound, but from the understanding of the improvisation technique, body dialogue, musical sensitivity and ginga also in the historical sense.*

Still in the field of Gymnastics, Locci et al. (2023) analyzed the role of the Hungarian immigrant coach Ilona Peuker as a protagonist in the development of Rhythmic Gymnastics (RG), also called Modern Gymnastics, in Brazil (1953 to 1995), especially regarding her role as a gymnastics coach, coach developer, and in the establishment of relationships between European and Brazilian cultures. They drew on oral histories and documentary research based on documents from Ilona (photographs, letters, certificates, etc.). From this study of images, it was possible to perceive the impact of an original style that enchanted audiences in Brazil and Europe due to the ‘Brazilness’ in Gymnastics.

Inspired by studies like this and based on previously developed research (Pasqua and Toledo, 2018; Pasqua and Toledo, 2019), we sought to understand Capoeira from image documents, which could give clues about its trajectories and the cultural and sporting impact on the practice of Capoeira in various fields of action and expression. Therefore, we emphasize studies on Capoeira iconography that have contributed to the cultural context of Capoeira (Pasqua, 2020; Pasqua et al., 2023; Araújo and Jaqueira, 2020; Castro Júnior, 2018, 2010; Desch-Obi, 2008).

As a polysemic bodily practice, Capoeira can be expressed in many dimensions. e.g., as an art, sport, history, or education tool. Authors from different backgrounds have interpreted Capoeira as an art and cultural manifestation (Silva, 2008; Castro Júnior, 2010; Tavares, 2012; Rosa, 2015; Reis, 1997; and Pasqua and Toledo, 2021), enhancing the body aspects of Capoeira. In sports, we mention Pasqua et al. (2012) and Gomes et al. (2025), who dealt with sports and Capoeira competitions, and Araújo and Jaqueira (2013), who conducted a praxeological analysis of the first regulated sport of Capoeira. For the historical facet, we highlight research developed by Soares (1993, 1998) and Pires (1996, 2001). For educational purposes, we mention Silva (2020a), Silva (2020b), and Silva (2009).

In order to understand the senses and meanings of Capoeira’s bodily knowledge, it is necessary to comprehend this manifestation beyond the Brazilian context, going overseas in the Black Atlantic scenario (Thompson, 2011b), as a cultural practice resulting from the African diaspora. Considering Capoeira as belonging to this African bodily substrate, or with a “wider range than Brazil” (Soares, 1993, p. 36), means admitting a more comprehensive stance for establishing parallels between Capoeira and other body manifestations considered ‘mothers’ and ‘cousins.’ In this way, we emphasize the African diaspora also from the perspective of the maps by Eltis and Richardson (2010).

There are bodily manifestations in Africa that are very similar to the practice of Capoeira in Brazil, such as N’golo and Bassúla, already mentioned by Soares (1993) and Pires (1996), which are known as the ‘mothers of Capoeira.’ The N’golo, focus of this manuscript, is also popularly known as the Zebra dance^2^. There are other dances that may have influenced the body knowledge repertoire of Capoeira, such as Bassúla, Cabangúla, and Umudinhú, mentioned in the study by Barão (1999), and the Cujuinha (warrior dance), Uianga (hunters’ dance), and the Cuissamba (dance of judgment and punishment), found with Zulu (1995).

**Figure 1:**
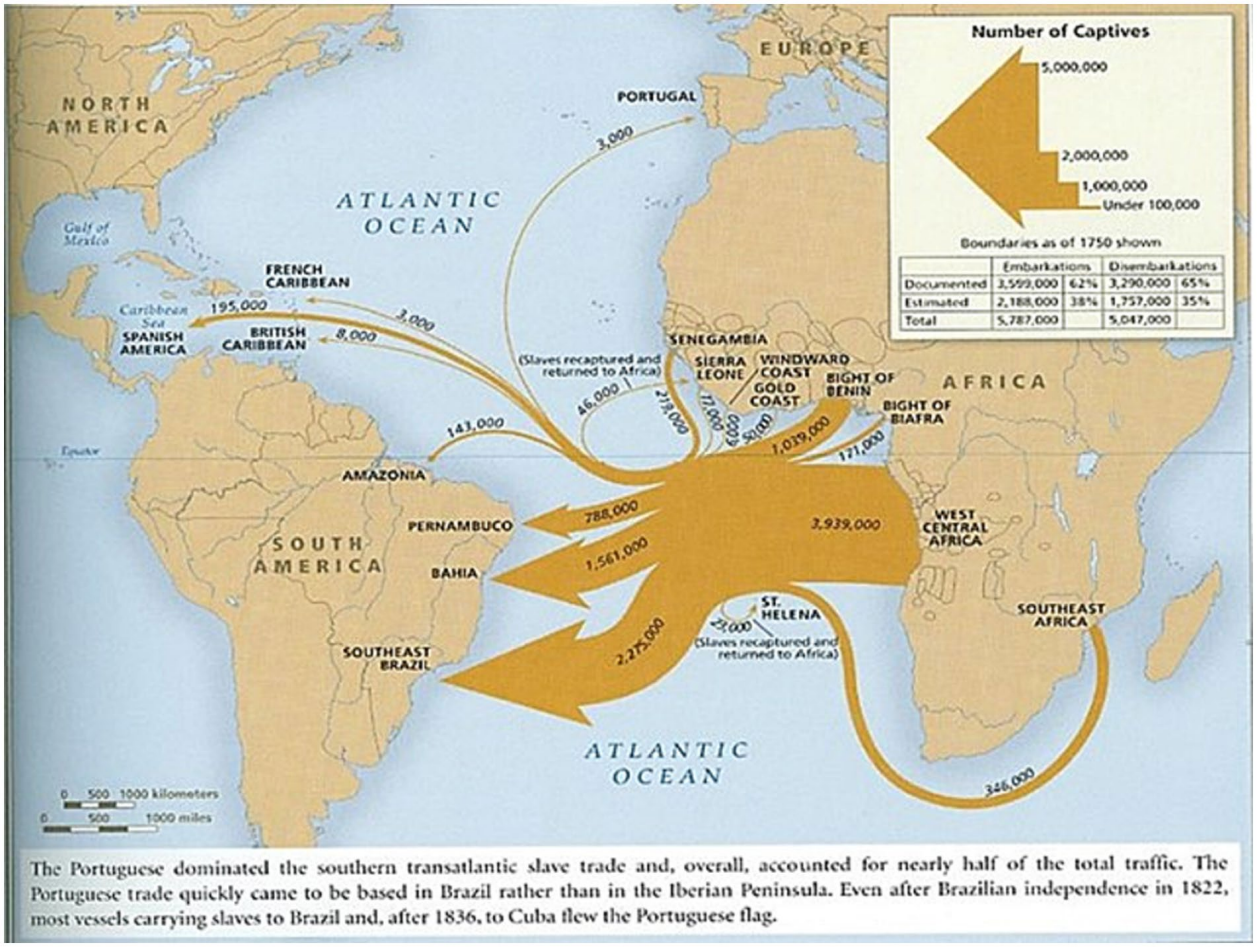
Map 1 (16). The Portuguese and Brazilian Transatlantic Slave Trade (1526–1867). Source: Eltis and Richardson (2010, p. 30).

Based on these premises, we present one of the maps in the book “Atlas of the Transatlantic Slave Trade,” by Eltis and Richardson (2010), two renowned historians who developed the first most comprehensive and updated atlas on the 350-year history of trafficking, kidnapping, and coercion of about 12.5 million people from Africa to almost all countries that had an Atlantic coast, 80% of trafficking trips ever made. This Slave Voyages^3^ database is available online and is one of the most complete sources of information on slavery in the Atlantic. We highlight the map of the Transatlantic Slave Trade from Portugal and Brazil in the period between 1,526 and 1867 (Eltis and Richardson, 2010, p. 30) to illustrate the number of people from different African geographic spaces (Figure 1).

According to Capoeira (2011), there are several “cousins” of Capoeira, bodily manifestations with African origin or due to the African presence in the Americas, in the *Black Atlantic* that currently have more visibility: stick fight (Haiti), jugar palo (Venezuela), calinda (Trinidad) maní or bambosá (Cuba), head-butting (Venezuela), knocking and kicking (United States), ladja (or danmyé, ronpom, or kokoyé, Martinique), mrengé (Comoros, Indian Ocean), morengy (or watsa) (Madagascar, Indian Ocean), and moringue (Reunion Island, Indian Ocean). Stotz and Falcão (2012) also dealt with laamb, a grappling fight present in Benin, Nigeria, the Ivory Coast, Togo, Ghana, Burkina Faso, and Mali.

In this context, Pasqua and Toledo (2021) point to the overcoming of a binary vision of Capoeira, strongly linked to fighting and games, and approaching the field of arts, not only by valuing the sphere of dance but also by what it *can* do for the body in the arts in general. Corroborating new conceptions of the body from Capoeira, like the *Capoeira-body* (Castro Júnior, 2010, p. 22), defined as “[…] *devices used for the narratives and knowledge production*” and a *“[…]corporeality that symbolizes the esthetic dimension of art-capoeira,*” something very specific of this corporal manifestation.

In other words, it is not the body in Capoeira or practicing Capoeira; it is a certain body, specific, that expresses itself (and therefore the artistic character) and that is constituted only while doing Capoeira, a body that ´*[..] besides being a fighter and a player, it is dancing and theatrical, creative, that is, a producing body of knowledge, free body, body as an identity that is at the same time collective (belonging to the universe of Capoeira) and preserves the individuality of the being.’* (Pasqua and Toledo, 2021, p. 89).

Departing from body knowledge to think about Capoeira, and based on the *esthetic of cool* (Thompson, 2011a), we would like to highlight two different (but complementary) esthetics from Capoeira-body narratives, such as *ginga* (Rosa, 2015) and *floreio* (Pasqua, 2020). Rosa defined *ginga* as, and subsequently, what the esthetics of *ginga* would be, as

*Ginga may be defined as a swaggering way of sliding or tilting (parts of) the body from one side to another when walking or otherwise, acting in society. It functions, more importantly, as a central mechanism with which one may “juggle” weight across time and space, while maintaining a cool and supple sense of flow.* (Rosa, 2015, p. 24)*.[…] this “juggling mechanism” is the key element of a non- hegemonic system of bodily organization and knowledge production, which exercises a distinct way of perceiving and interacting with others.*

Furthermore, based on Thompson (2011b) and Gottschild (1998), Rosa (2015) defines the esthetics of *ginga*, thought of from a polyrhythmic body, with the following principles: *(a) call-and-response and apartness in movement;* and *(b) serpentine pathways and high-affect juxtapositions,* in which it means (a) question and answer (dialogical relation) and isolation of the parts of the body in time and space (from the privilege of a particular form, a style to the detriment of a standard form) and (b) patterns in the form of a serpent, the movement like a snake; spiral; ability to move lightly in sinuous patterns) and high-impact juxtapositions (ability to throw one’s body into balance and imbalance—irony and paradox—juxtaposition also observed in movements that distance, delay, disorient, surprise or shock, with characteristics of fluidity, body flexibility, from swings, turns, rounds, circular, spiral movements.

On the other hand, Pasqua (2011, p.101) identified that one of the most unique characteristics of Capoeira, among many others, is the *floreio* (more than only acrobatic gestures), which can be understood as *‘[…] a bodily, factual and mysterious knowledge, expressed in a multifaceted way through movement and between movements, under a condition that carries the ancestry and presence in the game and that, therefore, allows negotiating with time and with each other.’* (Pasqua, 2020, p. 261). The acrobatic *floreios* in Capoeira inspire even more forms and creative possibilities of expression, such as breaking the body, falling apart, and melting, resulting in completely non-standardized forms, reinforcing this heritage of preservation of different identities and ways of moving in a cultural practice.

We assume that these body narratives present in Capoeira, such as the *ginga* and the *floreio,* have been shaped by a heterogeneous set of ideas under which African peoples and their descendants have recuperated and (re)structured their communities and systems of knowledge production in Brazil and other colonial places. Following this strand of thought, this process of heritage can be understood as a process of identification, as Rosa (2015, p. 7) stated:

*I pulled the expression “processes of identification” from the writings of Martín-Barbero (1987,^2003^). I use it here to differentiate “essentialized identities” (*i.e.*, fixed ideas, images, and discourses whose* var*iables are imagined static and constant) from flexible and complex processes articulated across a range of intersecting categories (gender, race/ethnicity, sexuality, class, nationality, etc.) situated in a particular time and space. I also dialogue with* Pinheiro’s (2008) *understanding of individuals as “movable territories.”*

In this sense, the goal of this article is to investigate and critically analyze a set of depictions of N’golo, one of the “mothers of Capoeira” (Capoeira ancestor), to understand the meanings of heritage representation in Capoeira. This discussion pays close attention to bodily narratives present in N’golo drawings and their impact on the collective imagination of this practice and consequent promotion of Cultural Diversity in sports nowadays.

With that in mind, our discussion pays close attention to how the *N’golo-bodies* are related to *Capoeira bodies* as devices of knowledge production based on heritage and how the understanding and appreciation of ancestry in bodily practices can promote the understanding of current forms of expression as well as respect for different identities currently present in the field of sport, reducing violence and prejudice and promoting social integration and a sense of humanity.

## 2 How to play in the *roda* (materials and methods)

This research is characterized as documentary with a historical approach, from the theoretical perspective of Revel (1998)^4^, which helps us synchronously identify and analyze the manifestations of body knowledge in different practices between the particular and the context of diaspora. This historical perspective was strengthened by the studies of Veyne (2008), which comprise the study of sources beyond the documents in order to understand the continuities and, above all, the discontinuities concerning this knowledge in the diasporic context.

The present research sought to understand the sources through a historical approach, and more specifically, through a narrative centered on the body, which was more coherent with the corporal knowledge of Capoeira. Thus, although Veyne (2008) mentions in his studies the appreciation of oral narratives (for a composition with documentary sources) in a historical study, we chose to revise this author’s proposal for body narratives. In this perspective, the appropriation of this author’s studies makes a fundamental contribution to identifying some continuities between these three chosen practices and, above all, the existing discontinuities between them, which together give Capoeira its identity. To understand the heritage of body narratives in Capoeira, we investigated one Capoeira ancestor practice, being our primary source being the N’golo drawings by Neves e Sousa.

To understand the body narrative present in the sources, we based ourselves on the theoretical framework of Castro Júnior (2010), based on his definition of *the Capoeira body*, a body that presents dramatic narratives:

*The body in capoeira is composed of a rich repertoire of gestures called blows, which represent a framework of ways with which the body performs movement. The blows are endowed with senses and meanings, and each one has a functionality for its practitioners. The constitution of this set of elements was conjectured over time in the practice of capoeira; new strikes appeared, preserving others and disappearing many others. In this cultural practice, there is a whole ritual of incorporating values that are transmitted by the masters through corporality that symbolizes the aesthetic dimension of capoeira art “what the body does is a socio-historical symbolization characteristic of each group.” (ibid., p. 23).*

Hence, the *Capoeira-body* concept already mentioned becomes essential to understanding an object of research. Thus, the analysis was performed from categories established *a priori* (Gil, 2007) (Table 1):

**Table 1:** Categories of the study.

Category	Description
Characterization of body practice	Was created in order to identify the body practice studied because they are not common in the field of Physical Education, being necessary to make yourself known, to know briefly about the history, its logic and objective.
musicality	Was created on account off understand and highlight the quality of percussive practices present in almost all artistic manifestations in the African central-west region influenced by Thompson’s studies (2011a)
Bodily elements.	Has with the objective of recognizing the gestures and dialoguing with the similarities and differences of the practices

Source: From the authors.

Because category 3 has the body as a centrality, we developed a form of identification that could facilitate the understanding of different forms of expression:

- Situations involving agonistic or fight-like movements (attack, opposition, combat, dispute)
- Situations involving dance-like movements (danced body modes, synchrony, body expression)
- Situations involving acrobatic movements (body inversion movements, balance, virtuosity)
- Situations involving displacement movements (gain of space, or exchange and transition of place)
- Situations involving theatrical movements (representation of a certain action or symbol of the culture of a character or person; dramatization).

For didactic and methodological purposes, we followed the procedures of analysis as well as Pasqua et al. (2023). In this study, the authors analyzed the drawings present in the illustrated book *Jogo da Capoeira* from Carybé’s (1951) work, paying close attention to a few overlapping game tactics or attitudes related to fight, play, dance, sports, and drama. In short, they argued that Carybé’s artistic depictions of Capoeira have been instrumental to the recognition of this Afrocentric practice as an Intangible Cultural Heritage of Humanity by UNESCO in 2014.

Our examination of Neves e Sousa’s drawings, as well as these authors’ works made with Carybé artworks, is grounded on theoretical tools and approaches drawn from the authors’ previous scholarship. At the start, our analysis is guided by fundamental elements of art criticism, namely *form, content*, and *context* (Belton, 1996), and its applicability to movement analysis (Banes, 2007), as well as Gumbrecht’s (2007). Understanding of the esthetic experience of sport spectatorship. Following Belton and Banes, we pay close attention to esthetic principles coming from the esthetics of the African diaspora.

Our analysis of *form* (the two-dimensional representation of N′golo’s esthetic principles and movement qualities on paper) and *content* (the multiple attitudes they elicit or evoke) is enhanced by an examination of the *context* of these N’golo drawings. Within this series of drawings, our analysis considers movements, body position, and dramatization. Meanwhile, our contextualization of the artist, the players, and the games seconds Castro Júnior’s understanding of the Capoeira-body, Lepecki’s (2010) notion of the *body as archive*, and Tavares’s concept of *weapon archive* (*arquivo arma*, in Portuguese Tavares, 2012). Together, these concepts enable us to unpack how the practice of Capoeira archives a particular way of thinking and doing things rooted in African heritage (N’golo) and how it also functions as a living weapon or act of resistance within a racist context. Hence, the importance of this visual collection.

This study was part of a larger research project, which investigated the practices whose primary sources were (A) N’golo drawings by Neves e Sousa, 1955; (B) Panther Dance (documentary video); and (C) Ladja (video recording—collection). To illustrate, we will present a summary of the main results in Table 2. However, in this manuscript, we will pay special attention only to N’golo.

**Table 2:** Summary table of analysis categories of some ancestral manifestations of Capoeira.

**Categories based on the *capoeira-body***			
	**N’golo**	**Panther dance**	**Ladja**
Characterization of body practice	Region: Angola African martial art Dispute—corporal dialogue of combat between two individuals—male. Form of circle. Only with clothes covering the lower body.	Region: Ivory Coast Ritual of the Senufo culture, African acrobatic dance. Dispute between clans—demonstration of skills—one individual—male. Form of circle. Panther outfit.	Region: Martinique Central American martial art with African roots. Dispute—body dialogue—combat between two individuals—male. Form of circle. Everyday or work clothes.
Musicality	Those responsible for the songs, in the standing position, also seem to practice fighting. It does not feature musical instruments. There are claps and mouth sounds, imitation of animal sounds (zebra and frog).	Those responsible for the instruments have a specific hat, play and sing in a sitting position, but do not seem to practice the dance. There are no claps. There is chorus response. Instrument similar to the *berimbau*, family of the *balafom*, in addition to the *xequerê (agbê)*	Those responsible for the instruments play and sing, in a seated position, but do not seem to practice fighting. There are no claps. There is chorus response. Instruments: drum - *tanbou alendjé*, played horizontally, “lying down”, with hand and foot, and on the back it is played with two *tibwa* sticks by another musician.
Bodily elements	Priority presents situations with agonistic or fight-like movements. There are situations with: dance-like; acrobatic; displacement and theatrical movements. Main bodily elements: swing, kicks (straight and rotated), defense, head butts, sweeps, takedowns and acrobatics, similarly to Capoeira gestures.	Priority presents situations with dance-like movements. There are situations with: acrobatics; displacements and theatrical movements. Mainly bodily elements: dance steps (similar to frevo and hip hop); acrobatics (similar to Capoeira and acrobatic gestures from Gymnastics and Dances). Serpentine pathways.	Priority presents situations with agonistic or fight-like movements. There are situations with: dance-like; acrobatic; displacement and theatrical movements. Main bodily elements: swing, kicks (straight and rotated), defense, head butts, sweeps, takedowns and acrobatics, similarly to Capoeira gestures.

Source: From the authors, based on Pasqua (2020) and Pasqua and Toledo (2018, 2019).

### 2.1 The primary source—N’golo drawings (Angola)

N’golo is an African manifestation considered the “mother” of Capoeira (Soares, 1993; Soares, 2002; Pires, 1996; Vieira and Assunção, 1998). Of Bantu origin, the N’golo comes from Angola, the region of greatest slave trade to Brazil. This is a collection of drawings by Neves e Souza, with drawings about N’golo. The imagery sample is composed of 20 drawings in black and white (the 20 drawings are numbered in the catalog from 9 to 27 and 33) of N’golo, present in the collection of the artist Albano Neves e Sousa, dated 1955.^5^

Albano Silvino Gama de Carvalho das Neves e Sousa was born in Matosinhos in 1921, during his parents’ vacation trip to Portugal. He lived his childhood in Angola, and later, he was integrated with the Mission of Ethnographic Studies of the Museum of Angola. Graduated in 1943 from the superior course of the Porto School of Fine Arts, he has his greatest importance in painting, but his work developed in different aspects: drawing, literature, botany, ethnology, etc., always portraying Europe, Brazil, and Africa, mainly Angola.

A thumbnail sample of the analyzed images is shown below. This diagram was organized according to the sequence in which the drawings were presented, having been numbered from 1 to 20 for this research, but they have specific numbers according to the organization of the folder in the museum. In this research they will be presented with the inscription, for example, Drawing NS 1 (9), indicating NS, which means *Neves e Sousa*; the first number, 1, indicating the sequence in which it was made available by the museum; and the number between parentheses, (9), indicating the number that is inscribed in the portfolio of drawings organized by the museum. Due to the limitations of the article, we will highlight only a few in a large size for the analysis of the results.

When it comes to ethical considerations about the source of Neves e Sousa’s drawings, the images were only provided and authorized for the exclusive purpose of this research, after consulting the collection in person, during a specific visit for this purpose, to the Livraria/Galeria Municipal Verney, in the municipality of Oeiras (Portugal) on 11 July 2018. Thus, the assignment and presentation of these images in the work were authorized by the Divisão de Biblioteca e Equipamentos Culturais—Município de Oeiras, Livraria/Galeria Municipal Verney/Colecção Neves e Sousa on July 24, 2018.

## 3 In the roda: *Capoeira-body* playing with *N’golo-*body (discussion)

As a matter of fact, the theme of Cultural Diversity has been gaining strength on the world stage, especially in the XX century, and it is relevant to international agendas, such as the UN 2030 Agenda (United Nation, 2015). Some of its sub-themes have been more prominent, such as debates on gender, ethnic-racial prejudice, ways of life of native peoples, ancestry, and immigration movements, among others, and have been studied by different areas, no longer only as a central theme of the Humanities (especially Anthropology), but constituting itself as a transversal theme of other areas, such as the area of Health, among them Sports Sciences. Thus, the theme of this research is aligned with this international trend and parallel with other studies that enable these theoretical approximations of Cultural Diversity and Sport.

We highlight the studies of Kamphoff et al. (2010), Gill (2017), and Gill and Kamphoff (2009), which have focused on the relationship between Cultural Diversity and Sports Psychology, evidencing the contemporary challenges that these relationships impose and should be considered in the psychological guidance of athletes and institutional sports programs in an applied way. Capoeira is in this context mainly because it is a polysemic practice and therefore also has a sporting character (Pasqua et al., 2012; Gomes et al., 2025), which carries in its practice aspects related to ancestry, cultural diversity, immigration, and the struggle of a people, among others.

Thus, studying this intersection between Capoeira, Cultural Diversity, and Sport contributes to its development in the cultural and sports fields, including its application in the context of Sports Psychology. According to the materials and methods, we will describe primary source analysis results referring to the ancestral bodily practices of Capoeira, following the categories established *a priori*.

### 3.1 Category 1. Characterization of body practice

N’golo is also known as Engolo or Zebra Dance (Assunção and Peçanha, 2008; Desch-Obi, 2008; Assunção, 2022; Kabwenha, 2024), a bodily-centered manifestation in which some blows resemble the kick of a zebra. This manifestation is typical of the Mucope region (Angola). In the work of Desch-Obi (2008), the author presents *Engolo techniques*. According to Assunção and Peçanha (2008, p. 16), “N’golo is danced by boys in southern Angola” as part of the puberty ritual, also called *mufico, efico,* or *efundula*, a rite of passage from the girl to the woman. The N’golo ritual is preceded by an open-hand fight, called Liuéta, in which you must hit the opponent’s head with your hands. Later, the N’golo, in which you must hit the opponent’s head with your feet, is performed, similar to a zebra kick.

Added to the N’golo collection are drawings of C’hancula, the manifestation of the Oxen dance, typical of the Mucope region (with the pastoral peoples), in which the subjects express in movements how their cattle are doing, what happened in the day, or other dangerous situations, the size of the ox, among others. The set of drawings by Neves e Sousa makes it possible to characterize the *N’golo-body* manifestation as a fight-dance practiced by male subjects, with attack and defense movements at all levels (high, medium, low) and with different strategies (blows from the inverted position) and the presence of acrobatic gestures.

There is a presentation of characters, the ones who perform the dance of the oxen and the Chipalanca, Mufico’s helper, which reveals an organizational structure of the ritualistic, since there is a highlight of a person, possibly a leader—the quimbanda. The mufico, a (feminine) rite of passage, is described by the theoretical framework in which only the male part is registered in the collection.

From the assumption of Castro Júnior (2010, p.22), which constitutes the term *Capoeira-body*, emphasizing it as a “body composed of a rich repertoire of gestures” (ibidem, p. 23), we think that this corporeality also plays a fundamental role in the understanding and study of certain practices. To do so, we depart from this same principle to create the terminology *N’golo-body*, understanding that this body narrates the social drama of those living in Mucope (Angola) in 1955, a culture based on rites of passage, celebrated in fight-dance, in a society that has at least one base of pastoral cultivation, with a repertoire of specific gestures that keep values incorporated and transmitted by the masters of N’golo, characterizing the esthetic dimension of this ritual.

### 3.2 Category 2. Musicality

In none of the 20 drawings in the sample are musical instruments, presenting only clues to the musicality in N’golo, such as, for example, sequences of movements that suggest rhythmic quality and gestures in which the two hands of the subjects touch each other, configuring palms, as can be seen in drawings NS 4 and NS 6.

According to our technical correspondent in Angola, Professor Janguinda Kambwetete Moniz (Kabwenha), the dispute in N’golo takes place in the form of a circle and is accompanied by clapping and sounds with the mouth, imitating the sound of animals, which in this case, he claimed to also be the sound of the animal frog Mafuma: ‘*The mouth sounds are sometimes songs composed in small verses…. Furthermore, most of the time, they have different intonations, it can be a whistle or a scream (all very melodic…). The sound that symbolizes the Frog (Mafuma) is incorporated right at the beginning of the practice’* (Professor Janguinda, via telephone contact, Oct. 2019).

He also claims that the berimbau is present in many places in the Angola region, with the names Mbulumbumba, Xikomba, Hungu, and Urucungo, but it is not part of the N’golo martial art. It is possible to find more nomenclatures of the berimbau in Rego (1968) in addition to specific studies on the instrument in Kubik (1979), which in no way relate this instrument to N’golo. As we also see in the documentary “Body Games: Capoeira and Ancestry” (Body Games, 2013), the berimbau is present in some regions of Angola; however, its direct relationship with N’golo is not evident.

Furthermore, in Sousa’s work ([1972?]), the author states, “The fight is rhythmic with applause and whoever makes a blow out of time is disqualified” (ibidem, s.n.). In this section, he emphasized the occurrence of palms, which he had already demonstrated in his drawing record. Clapping provides rhythmic quality, and it is not a musical instrument. This statement was also registered in De Araújo (2004).

In this way, we identified a continuity of the rhythmic quality of the clapping present in N’golo, also in Capoeira; however, there was a discontinuity in the use of the imitation of the sounds of the animals (frog and zebra) at the moment of the circle. In most Capoeira groups, there are songs, chants, *corridos*, and musical instruments, with no imitation of animal sounds to compose the “orchestra” of the circle.

### 3.3 Category 3. Bodily elements

**Figure 2:**
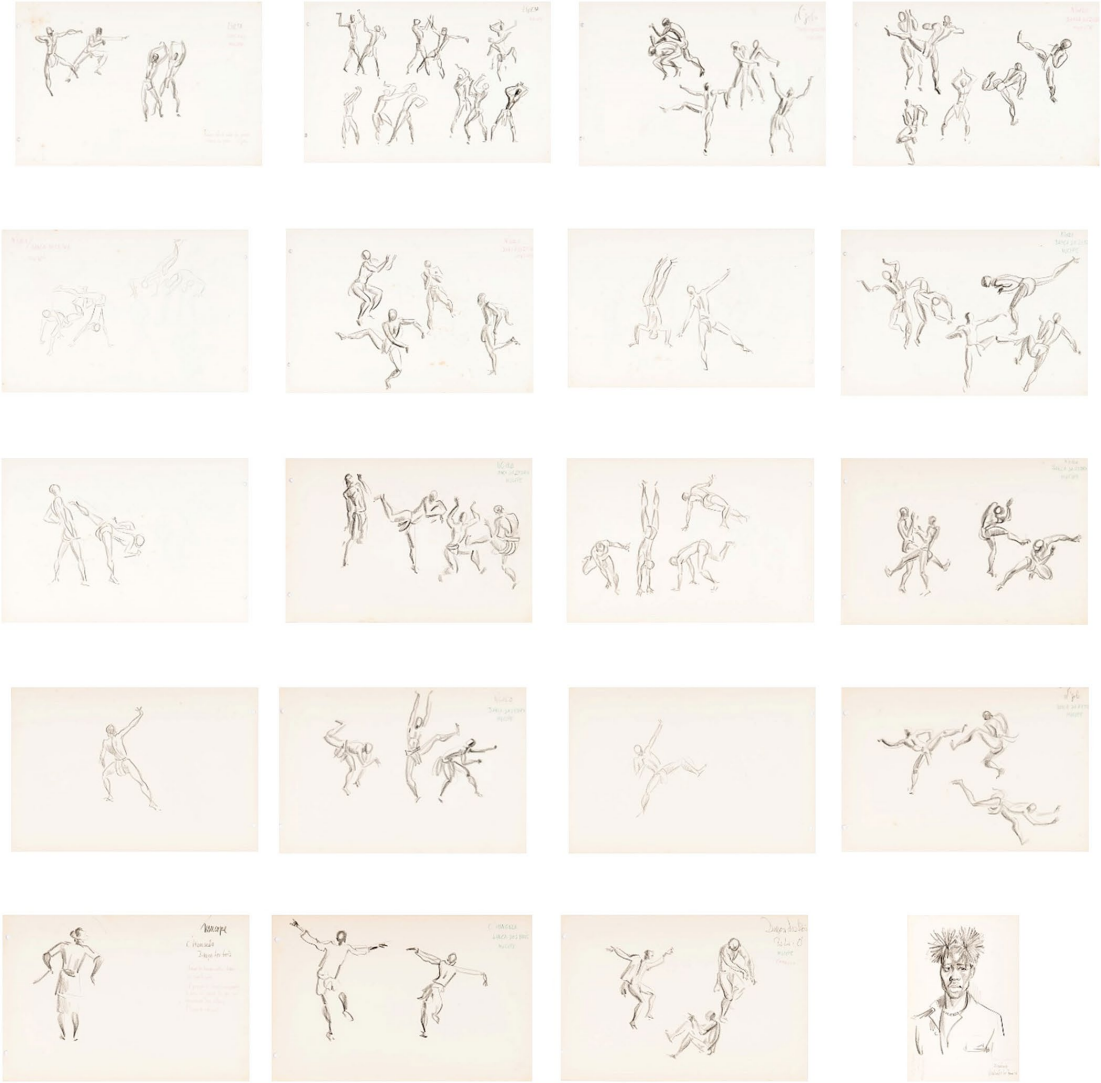
Diagram of N’golo drawings, Livraria-galeria Municipal Verney (2018). Source: Pasqua (2020, p. 130).

In N’golo, there are individual and double gestures with male subjects. We adopted as an analysis procedure only to bring the abbreviations of the drawings, as already mentioned (i.e., NS + number), which can be consulted in the diagram (Figure 2) or in some of the larger examples that we will demonstrate below. In general, we present what was identified in the sample in the table (Table 3).

**Table 3:** Analysis of situations present in N’golo Drawings of Livraria-galeria Municipal Verney (2018).

**Evidence**	**Quantity**	**Drawings**
Situations with only agonistic or fight-like movements	4	NS 2, NS 9, NS 10, NS 12
Situations with only dance-like movements	6	NS 1, NS 6, NS 13, NS 15, NS 18, NS 19
Situations with only theatrical movements	1	NS 20
Situations with only displacement movements	0	–
Situations with only acrobatic movements	0	–
Situations with only agonistic or fight-like movements and dance-like movements	5	NS 3, NS 4, NS 8, NS 14, NS 16
Situations with dance-like movements and acrobatic movements	1	NS 7
Situations with agonistic or fight-like movements, acrobatic movements and displacement movements	2	NS 5 e NS 11
Situations with dance-like movements and theatrical movements	2	NS 17 e NS 19

Source: From the authors, based on Pasqua (2020).

From the general analysis, we highlight here only one drawing of each situation.

#### 3.3.1 Situations involving agonistic or fight-like movements N’golo

**Figure 3:**
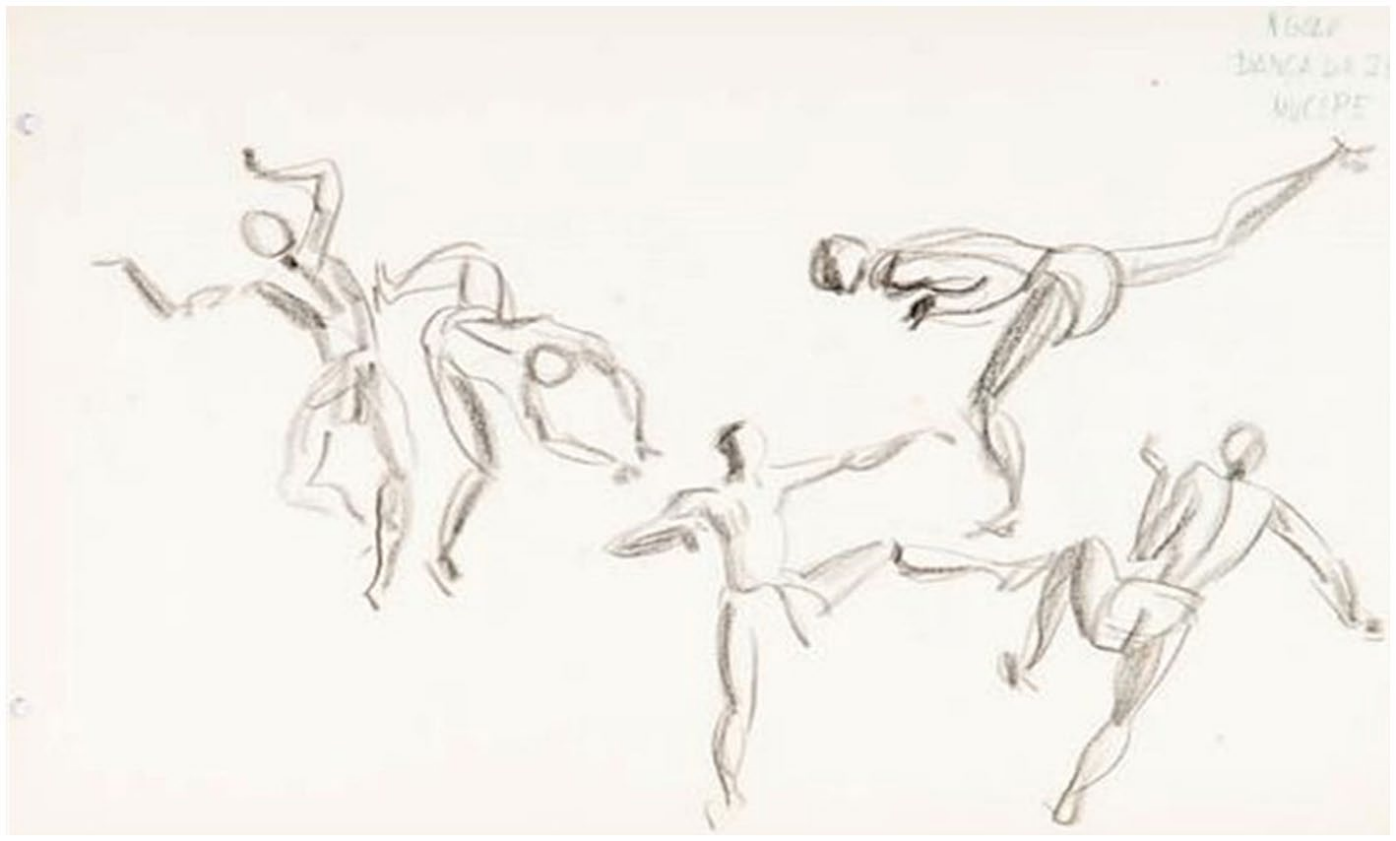
Drawing NS 8 (16)—N’golo—Dança da Zebra—Mucope. Source: Acervo Neves e Sousa. Livraria-galeria Municipal Verney (2018). In: Pasqua (2020, p. 150).

In Figure 3, there is a fight-like situation with agonistic movements in which the pairs vary between the high and medium plane positions. Consider the drawing divided into three parts: the first being double n.1 (one person standing and the other in an inverted position); the second pair being n.2 (both people are standing); and person n.3 (alone). In parts 1 and 2, there is an evident bodily dispute, and in part 3, it looks like a type of rehearsal or enhancement of a blow with the right leg back or a situation with balletic movement. In 2, the person strikes a kick with his left leg forward toward the opponent while the opponent seems to defend with his left knee, or it could also be that he is attacking with a *joelhada (knee kick)* at the same time as receiving the blow.

Still on part n. 1, in the work of Desch-Obi (2008, p. 221), it is possible to identify an image very similar to the one found in the collection, which describes how to *push kicks to the back* (*from an inverted position*). In Capoeira it is possible to find kicks backward from the inverted position by means of a more rectilinear movement, such as a *calcanheira* (heel kick), um *gancho* (kick with the outside side of the foot), um *pisão* (kick with the sole of the foot), or using a circular motion such as *meia-lua-de-compasso* or *rabo-de-arraia* (both blows performed in a rotating manner, attacking with the foot, whether or not using the hands on the ground).

#### 3.3.2 Situations with dance-like movements N’golo

**Figure 4:**
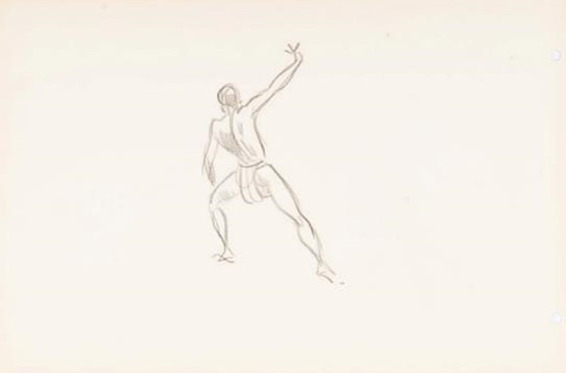
Drawing NS 13 (21) –s/título (reverso do n° 20). Source: Acervo Neves e Sousa. Livraria-galeria Municipal Verney (2018). In: Pasqua (2020, p. 158).

In Figure 4, there is a situation of dance-like movements. The person is in a high plane, supporting most of the weight on the front leg, which has the knee flexed, while appearing to support or even slide the left foot backwards. At the same time, his right arm is raised upwards, and his hands are with their fingers. This configuration is much more similar to a danced movement than a fought one; however, it refers to the *ginga* movement in Capoeira, the movement of *standing rasteira,* and *the* dance movements of the orixás (orishas). This drawing appears in the work of Desch-Obi (2008, p. 223) with the description *standing foot sweep.*

And we consider the limit of the source because the subject has its back turned, generating difficulty in identifying the subject’s expressiveness, whether it is aggressive or of celebration or contemplation. Why did the artist want to portray the subject from behind? What would your intention be? To portray the beauty or exoticism of these *N’golo-bodies?* Or would it be another form of bodily expression present in this manifestation, in addition to the fight, a pause, or a situation of balletic or theatricality that is very important in this context of the fight or of the ritual performance? If it is another form of expression that is more theatrical and dance-present in the fight, we point to a continuity of this *characteristic* and discontinuity for the *form* of this expression in Capoeira.

#### 3.3.3 Situations with acrobatic and displacement movements N’golo

**Figure 5:**
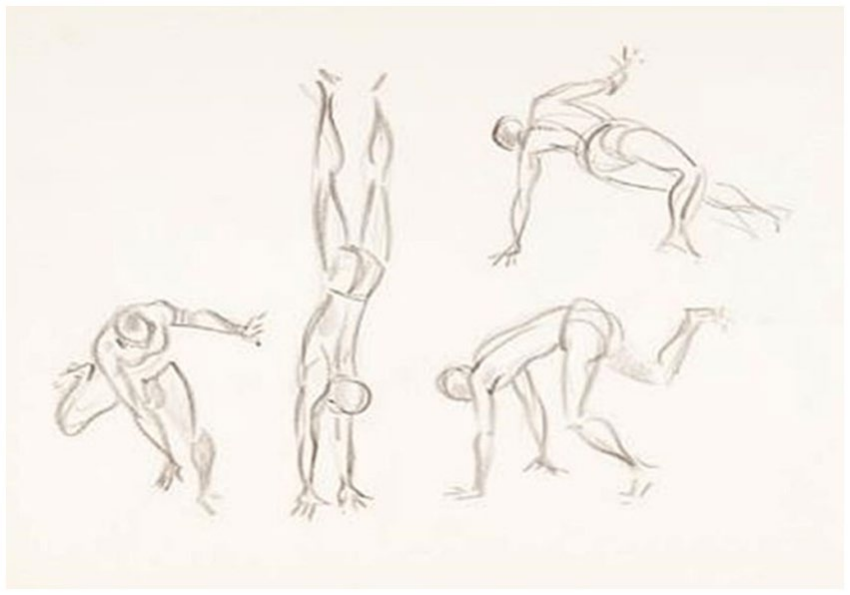
Drawing NS 11 (19) –s/ título (reverso do n° 18). Source: Acervo Neves e Sousa. Livraria-galeria Municipal Verney (2018). In: Pasqua (2020, p. 154).

In Figure 5, there is a situation of acrobatics, with displacement and possibly agonistic movements. There is a pair in the context of a dispute and two images with individual movements. This image appears in clippings in the work of Desch-Obi (2008, p. 219, 223, and 224). Consider the image divided into 3 parts, with double no. 1, the person with both hands on the floor (no. 2), and the person with one hand on the floor and the other hand suspended (no. 3).

From the reference drawing NS 11, there is, in item 1, a subject who is in inverted support, which would be similar to the *bananeira* (handstand) of Capoeira, and the other subject is in the middle plane, with all his weight on his left leg, knees flexed, arms alternating, looking at his partner, which in Capoeira would suggest a counterattack of *cabeçada* (headbutt). Desch-Obi (2008, p. 219) described the same drawing as in 1. *Lunge with hand on the ground*, a charge, and a *bote* (boat) with its hand on the ground, and 2. *Handstand.*

In item 2, it is possible to identify a subject in a low plane, with both hands resting on the ground, practicing a backward blow from the back position. In Capoeira, this could be described as a *calcanheira* (rectilinear motion), a *meia-lua-de-compasso,* or *rabo de arraia* (blows from circular movements). Desch-Obi (2008, p. 223) described drawing as *a circular kick with the body supported by hands.* It can also be interpreted as an essay on *aú* (cartwheel), an acrobatic *floreio*.

In item 3, the person appears to move on the ground in a circular shape with the entire weight supported by the left arm and hand, which resembles the movement of *rolê* da Capoeira (passage on the floor rolling). These movements are performed in order to gain space or to move closer to study the space of the floor and other objects. Compared to Laban’s factor of movement *space*, one could think of the study of space through the kinsphere of one and the other. Desch-Obi (2008, p. 224) describes drawing as *lateral movements out of low defensive postures.*

In all the items observed in Figure 5, we identified continuities in relation to Capoeira in all situations, whether of attempted attack during the execution of the acrobatics or of displacement and study of space on the floor.

#### 3.3.4 Situations with theatrical movements N’golo

**Figure 6:**
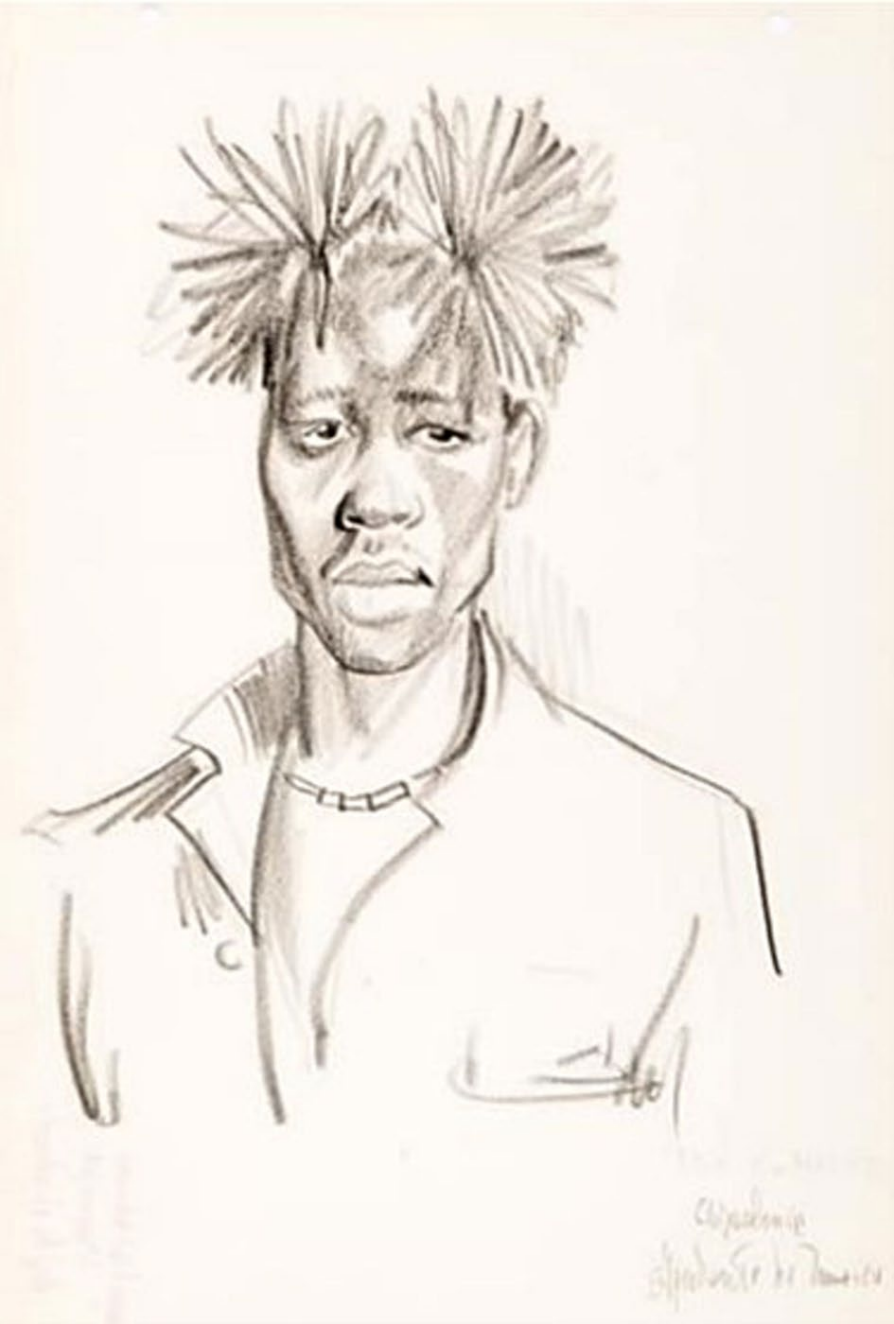
Drawing NS 20 (33)—Dança dos bois—Tala-Ó—Mucope—Cankula. Source: Acervo Neves e Sousa. Livraria-galeria Municipal Verney (2018). In: Pasqua (2020, p. 167).

In Figure 6 there is a theatrical situation, in this case, the highlight of the character, in which a subject described in the drawing as Chipalanca, Ajudante do Mufico (Mufico’s Helper), is represented. As previously described, the Mufico is the rite of passage from girl to woman in which the practice of the martial art Engolo is practiced.

When resuming Figure 2, Diagram of N ´golo drawings Livraria-galeria Municipal Verney (2018), the last 4 drawings, NS 17, 18, 19, and 20, are characterized by theatrical situations, with NS 17 to 20 identified as “Dança dos Bois” (Dance of the oxen) and NS 20 as “Chipalanca.”

Unlike the other drawings, the character here comes to life through the details and expressions of the face. There is a similar drawing in another work by the same author (Neves e Sousa, 1972 [?], n.p.). It is noted that the oldest record (1955) relates the “Dança dos Bois” to the character “Chipalanca,” and later, in 1972[?], the author relates the same character to the N ´golo, which possibly indicates the close relationship between “Dança dos Bois” and N ´golo.

There is a drawing extremely similar to this, one being a reference to the other, in the work of Neves e Sousa (1972 [?], n.p.) with the following caption: *“This is Chipalanca <<Quimbanda> > which governs the ceremonies of the <<Efico> > in Mucope and which, therefore, also gives laws concerning the N’golo… In short, he is the <<Mestre Pastinha> > of this function…*. “Approximately 20 years after his first drawing, the author establishes the relations between the master of the N’golo ritual and the Capoeira master. Mestre Pastinha was a recognized Capoeira master in Brazil during the twentieth century.

It should be noted that the name of this master Chipalanca is also a reference to Quimbanda, which is the name of an Afro-Brazilian religion, and in this way, we could infer that he would be a master Curandeiro (healer). In Brazil, there are three religions of African origin, often taken as equals, such as Candomblé, Umbanda, and Quimbanda. Despite their similarities, they were labeled as right–left, white-black, due to the repression of Christian morality (Carvalho and Bairrão, 2019).

The term Curandeiro has other meanings, which are as follows:

*Curandeiro, *s.m.* Otyimbanda / Various species of … omuhakulf, omufili, ofilai, enkhununu (in the case of alleged greater magical powers), ondundu (idem), embanda (idem) / Antelope horn used by the … Ohiva (and more meanings of onomatopoeia).

*Curandice, *s.f.* Oumbanda, oundundu, oufiliai ounhkhunun/Payment due by … Omalilo, Ondyambi. (Silva,1966, p. 145).

The term “Curandeiro,” we have in Kubik (1979, p. 25):

*Umbanda* is the indigenous medical science of Angola. Because its concepts are based on theories about human nature, unlike Western cultures, *Umbanda* has often been misinterpreted by Westerners as “witchcraft.” The *vimbanda* (plural of *kimbanda*) were alternately called “priests,” “soothsayers,” “witches’ doctors,” “prophets,” “healers,” “native doctors,” “herbalists,” etc., according to the philosophies and religious perspective of the observer.

Therefore, it is possible to suppose that Neves e Sousa gave some prominence to this character because of his important role in the ritual of the N’golo, which is circumscribed to a rite of passage and has a religious nature.

On the occasion of the visit to the Neves e Sousa Collection, when the request for the folders of drawings referring to the N ´golo was made, we were unaware that there would be the “Dança dos Bois” in the same folder, which was a revelation about the complexity of the practices of the N ´golo. It should be noted that the N ´golo presents not only a martial art but a more complex ritual in which the Engolo fight is part.

According to researcher Janguinda Kambwetete Kabwenha (corresponding Angolan author), in the festivals and rituals of the N ´golo, the aforementioned C ´Hancula may or may not occur, whose correct spelling is Onkhakula (Neves e Sousa leaves in his register two ways of writing that should probably help in the pronunciation—C ´Hancula and Cankula, but which would not necessarily be the correct ones). Onkhakula is also a ritual of dance and fighting, ox poetry, or cattle poetry, because they are pastoral regions, according to ethnographic studies conducted in this region by missionary Father Carlos Estermann.

Professor Josivaldo Pires de Oliveira (Mestre Bel), a scholar of the sources and works of the Spiritan missionary priest Carlos Estermann, in southern Angola, 1924–1976 (Oliveira, 2018), states about the importance of the studies of these missionaries with the objective of evangelization in Angola, because, despite the colonialist objective of knowing the culture of the so-called “indigenous” in depth so that they can penetrate their universe of representation and bring salvation, they have made a great contribution to science:

*These societies practiced pastoralism, and as they still practice today, the cultural dimension of the pastoral life of these populations did not escape Father Estermann’s descriptions: music, art, religion, etc. For these societies, cattle were an essential element of social life, related to elements of economic, political, religious, and customary nature. The records produced by the priest did not miss these elements of characterization of the groups that inhabited the south of Angola, more precisely, the southwestern region. Some of these practices, which emphasized the ritual value of cattle, attracted his attention, resulting in important records in his relevant ethnographic essays, published at different times during his missionary activity. In this way, it can be said that Father Carlos Estermann was a Spiritan missionary who dedicated himself to the ethnography of the pastoral peoples and cultures of southern Angola.* (Oliveira, 2018, p. 52).

#### 3.3.5 Considerations of category 3 bodily element

In view of the presentation of the drawings, it is possible to conclude that the ritual of the N’golo comprises other dances and fights besides the Engolo (zebra dance), such as the Liuéta (which precedes the Engolo) and the Onkhakula (Dança dos Bois—Dance of the oxen), and the religious rites based on the figure of the Curandeiro (healer). In this way, we point to a discontinuity in relation to Capoeira because the practice of the latter is not linked to any rite of passage present in society.

The rites of passage in Capoeira take place in the Baptisms and Festivals, in which the subject changes his rank, color of the rope/cord/cordel, or just the title, much more due to a sporting tradition resulting from modernity in Brazil than a mention of the transformations of the human being in his life cycles. Furthermore, the Capoeira schools in general do not conceive the ritual of the Capoeira roda (circle) as a specific religion but rather, much more in the sense of a philosophy of Capoeira, in which each capoeirista (Capoeira player) has their own.

There is no record of the presence of the female sex, even though the event of Mufico is a ritual of the passage from girl to woman, a celebration of the moment of puberty. The female part of the rituals is not revealed, or little is portrayed, possibly because it is more reserved. In Capoeira, the presence and performance of the female sex is verified and has been highly valued, especially in recent years, with the formation of several Capoeira masters, in addition to specifically female events, which do not occur with the practice of fighting in the N’golo. In this case, the female presence in the source of the drawings is not identified and is not even today, because there is no evidence of N ´golo masters in remaining places of the practice, as could also be observed in the documentary “Jogo de Corpo: Capoeira e Ancestralidade” (2013).

This visual aspect of the *N’golo-body*, analyzed first-hand without any interference from literature by itself, for Capoeira connoisseurs would be enough to establish relationships of extreme similarity. The source reinforces the relationship of similarity and awakens us to think of the *Capoeira-body* (Castro Júnior, 2010) as one of the possible legacies of an *N’golo-body.*

In view of the analysis of the drawings, although the majority presented were situations of agonistic movements and situations of danced movements, confirming the fight-dance binomial, we reinforce that it was possible to advance as well as in the study of Capoeira, understanding that N’golo is also about a polysemic practice. From the analysis of the collection, it was possible to identify that the N’golo comprises other dances and fights besides the N’golo (zebra dance), such as the Liuéta (which precedes the N’golo) and the Onkhakula (oxen dance), and the religious rites based on the figure of Chipalanca, healer (Figure 6).

The power of the *N’golo-body*, based on a body narrative of a series of movements that sometimes resemble fighting and sometimes resemble dancing, which is unbalanced forward and backward and sideways, a conception of balance and imbalance, as well as the polycentric and polyrhythmic body of Rosa (2015), with her pelvic and shoulder girdles talking in different directions, with blows, defenses, dances, displacements, and acrobatic movements expressed in inverted positions, in addition to characters such as the subject of the dance of the oxen and the Chipalanca, representing the rituals of the people of Mucope. Moreover, this pugilistic tradition observable in the drawings, as well as in the assumptions of Desch-Obi (2008), brings the *N’golo-body* closer to the body-archive-weapon conception of Tavares (2012), a tradition of fighting in Liuéta, in N’golo, and in C’hankula.

N’golo-body also plays *floreios* in nouns and verbs (form and characteristic, Pasqua, 2020). The *floreio,* in terms of the quality of form and acrobatic gesture, appears in inverted support movements, such as the aú, headstand, head spin, and handstand. In addition, it can be thought of as a *luta floreada* (‘flowery fight,’ ‘decorated fight,’ and ‘beautiful fight’), just like Capoeira, because it has different situations with danced movements during the dispute, since many times they were also portrayed in drawings with fight situations, just as there are several drawings with only situations of danced movements. The *floreio,* as a characteristic, also unfolds in the dimension of allegory in the dance of the oxen, as the body expression performed in the dance is allegorical, a metaphor for everyday events expressed in the language of the body that dances. The source reinforces the relationship of similarity and prompts people to think of the *Capoeira-body* (Castro Júnior, 2010) as one of the possible legacies of an *N’golo-body.*

Furthermore, the *floreio,* as a form, has a dimension of identity in the N ´golo, whether with the dimension of mandinga in the unfolding of dispute or *agon* (NS 5), a situation of play or setting up an ambush or trap, or *mimicry* (NS 7 and NS 11). The *floreio* as a characteristic also unfolds in the dimension of allegory in the Dança dos Bois because the body expression performed in the dance is allegorical and a metaphor for everyday events expressed in the language of the dancing body.

## 4 Ending the roda or maybe opening a new one… (conclusion)

As previously mentioned, this study opens new perspectives to consider Capoeira in the sportivized scope, considering it as a polysemic practice that addresses dance, game, fight, and sport. This study presents analyses of the cultural relationship between the bodily practices of different peoples and highlights the similarities and differences between them. In this perspective, it identifies the influences of N’golo in the current practice of Capoeira, as well as its differences, arising from a miscegenation of races and peoples, and in the recovery and reinvention of knowledge in the context of the struggle and resistance experienced in colonial Brazil (slavery process). These cultural and historical aspects cannot be neglected from their current practice and should be considered not only in the field of sociology or anthropology of sport but also in other areas, such as sports psychology.

Based on the assumption that Capoeira is a polysemic practice and that it must be understood beyond the Brazilian context, we seek a foundation in the body knowledge of African matrices that were recovered and reinvented overseas. From the study of African body practices considered ancestral to Capoeira, specifically the N’golo in this manuscript, we were able to infer that the body narratives present in the *N’golo-body* are very similar to the *Capoeira-body*, expanding the understanding of N’golo beyond fighting as a multifaceted practice. Therefore, the polysemic characteristic of Capoeira also seems to be a reflection of the polysemy present in N’golo.

Despite the discontinuities in relation to the musicality of the N’golo, the religious aspect and the figure of the healer related to the Efico, and the feminine absence of fighting practices, the *floreio* manifests itself in the N’golo in a very similar way to Capoeira, both as a form and characteristic, in the dimensions of allegory, identity, and mandinga. As in Capoeira, the *floreio* and *ginga* are bodily knowledge of the N’golo, imprinting similar stylistic characteristics.

In a paradoxical way, the analysis itself pointed to components that are not visible, or intangible, enigmatic units that are difficult to understand, because it is something that is felt and accomplished bodily, which in turn gave rise to a more fluid analysis, not being the objective of this article, which will be presented in future work, based on a different background, the Anthropology of Performance (Turner, 1988). In this sense, the aspect of intangibility of the floreio will be analyzed, unfolding in looks at the worldview, the becoming-animal, the weapon, the spiritual and the anthropophagic behavior, and the mystery understood under allegories.

For future research, we will continue to analyze images about other ancestral practices of Capoeira, namely, one of non-Bantu origin, which historically had a minority of enslaved people brought to Brazil from the Ivory Coast, the manifestation called Panther Dance, and one of a similar context to the process of recovery and reinvention of knowledge in the Americas, specifically in Martinique, French Caribbean, the manifestation called Ladja, considered one of the “cousins” of Capoeira.

In addition, we intend to continue investigating other ancestral practices that can provide clues about the esthetics present in *Capoeira-bodies* today, which impact the understanding of the practice itself as well as the understanding of Cultural Diversity and Ethnic-racial relations in sports in Brazil and around the world.

Thus, admitting the potency of *N’golo-bodies* and *Capoeira-bodies* as devices for knowledge production, leaving behind the need for classification, and exploring the senses and meanings of the educational practice of Capoeira as a representation of Cultural Diversity in sports is what we envision also broader understanding for promoting inclusion by respecting ancestry and sense of humanity belonging. This research showed that, from different theoretical epistemologies (Capoeira framework, history, and body-Capoeira narratives), we can consider Capoeira as a polysemic bodily manifestation that dialogs and transits, which is and manifests itself in a plural way at the same time as in unity, dance, game, fight, art, and culture.

The research data show and strengthen a perspective of African influence, already so debated in Brazil (and outside of it), but it brings to the center of the debate how Capoeira can be a practice that, at the same time, has a cultural diversity and also produces a unique culture, which is on the move in history (with cultural backgrounds). Re-signifying, therefore, the ways of understanding, practicing, and living the bodily manifestations that have cultural diversity in their genesis from their teaching and what their learning can potentiate for the formation of more sensitive, non-binary, open citizens of the world to the experience, to the ecological and human conceptions of each of these cultures, producing a re-signification of themselves and of society.

## Permission to reuse and copyright

Permission was obtained for the use of copyright for the use of N’golo de Neves e Sousa drawings assigned exclusively for the purposes of this research by Dr. Gaspar Matos (Division Head) and Dr. Fernanda Marta Marques (Superior Technique), both from *Divisão de Biblioteca e Equipamentos Culturais—Município de Oeiras, Livraria/Galeria Municipal Verney/Colecção Neves e Sousa,* referring to the Ph.D. thesis financed by the Brazilian Government—Coordination for the Improvement of Higher Education Personnel—Brazil (CAPES).

## Data Availability

The original contributions presented in the study are included in the article/supplementary material, further inquiries can be directed to the corresponding author.

## Footnotes

^1^For illustration only, it consists of a circle with different people, in which someone is placed around the circle holding Capoeira musical instruments, like *berimbau* (resonating gourds), *atabaque* (drum), *pandeiro* (tambourine), and *agogô* (double bell), while people are clapping and singing. Inside the circle are two people playing—fighting and dancing—according to the melody. Each different kind of berimbau sound means varied types of Capoeira games.

^2^Our corresponding author from Angola, Janguinda Kambwetete Moniz (Professor Cabuenha), an artist and Capoeira player, gave us the information that it is not just an allusion to the zebra animal but also to the Mufic frog.

^3^*Slave Voyages—TRANS-ATLANTIC SLAVE TRADE DATABASE.* Available at https://www.slavevoyages.org/.

^4^From particularity to universality, the scale games represent an experience of microanalysis.

^5^Source for bibliographic data on the artist Neves e Sousa: insert of the Livraria-galeria Municipal Verney (2018). Edition of Municipality of Oeiras (Portugal). Division of Culture and Tourism, with the collaboration of Professor Dr. Fernanda Marta Marques. The images were only given and authorized for the exclusive purposes of Ph.D. research (Pasqua, 2020).
